# Supplementary material for: Evidence Based Gait Analysis Interpretation Tools (EB-GAIT) treatment recommendation and outcome prediction models to support decision-making based on clinical gait analysis data
Source: PLoS One. 2025 Jul 29;20(7):e0328036. doi: 10.1371/journal.pone.0328036 (PMC12306754; doi:10.1371/journal.pone.0328036)
Supplement: Appendix S1 — (DOCX) [file pone.0328036.s001.docx]

# Appendix 1 - Treatment Recommendation Model Features

**Neural Rhizotomy**: Era, Age, Diagnosis, Adductor Spasticity, Hamstring Spasticity, Plantarflexor Spasticity, Rectus Femoris Spasticity, Net Dimensionless Oxygen Consumption (% Typ), Dynamic Motor Control (Walking), Affected Side, Prior Neural Rhizotomy, Initial Contact Knee Angle Sagittal Plane, Initial Contact Ankle Angle Sagittal Plane, Maximum Stance Ankle Angle Sagittal Plane, Mean Swing Ankle Angle Sagittal Plane, GMFCS

**Rectus Transfer**: Era, Age, Diagnosis, Rectus Femoris Spasticity, Affected Side, Prior Rectus Femoris Transfer, Prior Neural Rhizotomy, ROMSwing Knee Angle Sagittal Plane, Initial Contact Knee Angle Sagittal Plane, Foot Off Knee Angle Sagittal Plane, Maximum Swing Knee Angle Sagittal Plane, GMFCS

**Psoas Lengthening**: Era, Age, Diagnosis, Popliteal Angle (Unilateral), Affected Side, Maximum Hip Extension, Prior Psoas Release, Mean Stance Pelvis Angle Sagittal Plane, Minimum Stance Pelvis Angle Sagittal Plane, Minimum Stance Hip Angle Sagittal Plane, Maximum Stance Pelvis Angle Sagittal Plane, GMFCS

**Hamstrings Lengthening**: Era, Age, Diagnosis, Maximum Knee Extension, Popliteal Angle (Unilateral), Affected Side, Prior Hams Lengthening, Initial Contact Pelvis Angle Sagittal Plane, Initial Contact Hip Angle Sagittal Plane, Initial Contact Knee Angle Sagittal Plane, Minimum Stance Knee Angle Sagittal Plane, Maximum Swing Semimembranosus Length, GMFCS

**Adductor Lengthening**: Era, Age, Diagnosis, Maximum Hip Abduction (Knee Extended), Maximum Hip Abduction (Knee Flexed), Hip Abductor Strength, Affected Side, Prior Adductor Release, Initial Contact Hip Angle Coronal Plane, Maximum Stance Hip Angle Coronal Plane, Minimum Swing Hip Angle Coronal Plane, GMFCS

**Calf Muscle Lengthening**: Era, Age, Diagnosis, Maximum Ankle Dorsiflexion (Knee Extended), Maximum Ankle Dorsiflexion (Knee Flexed), Plantarflexor Spasticity, Plantarflexor Strength, Weightbearing Foot Position, Weightbearing Foot Position Severity, Affected Side, Prior Gastroc Soleus Lengthening, Initial Contact Knee Angle Sagittal Plane, Initial Contact Ankle Angle Sagittal Plane, GMFCS

**Femoral Derotation Osteotomy**: Femoral Torsion, Era, Age, Diagnosis, Bimalleolar Axis Angle, Maximum External Hip Rotation, Maximum Internal Hip Rotation, Affected Side, Prior Femoral Derotation Osteotomy, Mean Stance Pelvis Angle Transverse Plane, Mean Stance Hip Angle Transverse Plane, Mean Stance Knee Angle Transverse Plane, Mean Stance Foot Angle Transverse Plane, Minimum Swing Knee Angle Coronal Plane, Maximum Swing Knee Angle Coronal Plane, GMFCS

**Tibial Derotation Osteotomy**: Femoral Torsion, Era, Age, Diagnosis, Bimalleolar Axis Angle, Maximum External Hip Rotation, Maximum Internal Hip Rotation, Affected Side, Prior Tibial Derotation Osteotomy, Mean Stance Pelvis Angle Transverse Plane, Mean Stance Hip Angle Transverse Plane, Mean Stance Knee Angle Transverse Plane, Mean Stance Foot Angle Transverse Plane, Minimum Swing Knee Angle Coronal Plane, Maximum Swing Knee Angle Coronal Plane, GMFCS

**Distal Femoral Extension Osteotomy + Patellar Advancement**: Era, Age, Diagnosis, Extensor Lag, Maximum Knee Extension, Patella Alta, Affected Side, Prior Hams Lengthening, Prior DFEO, Prior DFEO + Patellar Advance, Prior Patellar Advance, Mid-Stance Knee Angle Sagittal Plane, GMFCS

**Patellar Advancement**: Era, Age, Diagnosis, Extensor Lag, Maximum Knee Extension, Patella Alta, Affected Side, Prior Hams Lengthening, Prior DFEO, Prior DFEO + Patellar Advance, Prior Patellar Advance, Mid-Stance Knee Angle Sagittal Plane, GMFCS

**Foot and Ankle Bony Reconstruction**: Era, Age, Diagnosis, Non-Weightbearing Arch, Non-Weightbearing Midfoot Motion, Weightbearing Forefoot Varus/Valgus, Weightbearing Forefoot Varus/Valgus Severity, Weightbearing Forefoot Ab/Adduction, Weightbearing Forefoot Ab/Adduction Severity, Weightbearing Foot Position, Weightbearing Foot Position Severity, Weightbearing Midfoot Position, Affected Side, Prior Foot and Ankle Bone, Prior Foot and Ankle Soft Tissue, Mean Stance Foot Angle Transverse Plane, GMFCS

**Foot and Ankle Soft Tissue Reconstruction**: Era, Age, Diagnosis, Non-Weightbearing Arch, Non-Weightbearing Forefoot Varus/Valgus, Non-Weightbearing Forefoot Varus/Valgus Severity, Non-Weightbearing Hindfoot Varus/Valgus, Non-Weightbearing Hindfoot Varus/Valgus Severity, Non-Weightbearing Midfoot Motion, First Ray Plantarflexion, Weightbearing Forefoot Varus/Valgus, Weightbearing Forefoot Varus/Valgus Severity, Weightbearing Forefoot Ab/Adduction, Weightbearing Forefoot Ab/Adduction Severity, Weightbearing Foot Position, Weightbearing Foot Position Severity, Weightbearing Midfoot Position, Affected Side, Prior Foot and Ankle Bone, Prior Foot and Ankle Soft Tissue, Mean Stance Foot Angle Transverse Plane, Mean Swing Foot Angle Transverse Plane, GMFCS
