# Supplementary material for: Evidence Based Gait Analysis Interpretation Tools (EB-GAIT) treatment recommendation and outcome prediction models to support decision-making based on clinical gait analysis data
Source: PLoS One. 2025 Jul 29;20(7):e0328036. doi: 10.1371/journal.pone.0328036 (PMC12306754; doi:10.1371/journal.pone.0328036)
Supplement: Appendix S2 — (DOCX) [file pone.0328036.s002.docx]

# Appendix 2 - Outcome Prediction Model Features

**Maximum Ankle Dorsiflexion (Knee Extended)**: Age, Maximum Ankle Dorsiflexion (Knee Extended), Maximum Ankle Dorsiflexion(Knee Flexed), Initial Contact Knee Angle Sagittal Plane, InitialContact Ankle Angle Sagittal Plane, Minimum Stance Knee Angle SagittalPlane, Maximum Stance Ankle Angle Sagittal Plane, Minimum Swing AnkleAngle Sagittal Plane, GMFCS, Interval Adductor Release, Interval DFEO +Patellar Advance, Interval Femoral Derotation Osteotomy, Interval Foot andAnkle Bone, Interval Foot and Ankle Soft Tissue, Interval Gastroc SoleusLengthening, Interval Hams Lengthening, Interval Neural Rhizotomy, IntervalPatellar Advance, Interval Psoas Release, Interval Rectus Femoris Transfer,Interval Tibial Derotation Osteotomy

**Maximum Knee Extension**: Age, Extensor Lag, Maximum Knee Extension, Patella Alta, Mean Stance KneeAngle Sagittal Plane, Minimum Stance Knee Angle Sagittal Plane, GMFCS,Interval Adductor Release, Interval DFEO + Patellar Advance, IntervalFemoral Derotation Osteotomy, Interval Foot and Ankle Bone, Interval Footand Ankle Soft Tissue, Interval Gastroc Soleus Lengthening, Interval HamsLengthening, Interval Neural Rhizotomy, Interval Patellar Advance, IntervalPsoas Release, Interval Rectus Femoris Transfer, Interval Tibial DerotationOsteotomy

**Trochanteric Prominence Angle**: Age, EOS Femoral Anteversion, EOS Bimalleolar Axis Angle, TrochantericProminence Angle, Bimalleolar Axis Angle, Maximum External Hip Rotation,Maximum Internal Hip Rotation, Mean Stance Pelvis Angle Transverse Plane,Mean Stance Hip Angle Transverse Plane, Mean Stance Knee Angle TransversePlane, Mean Stance Foot Angle Transverse Plane, GMFCS, Interval AdductorRelease, Interval DFEO + Patellar Advance, Interval Femoral DerotationOsteotomy, Interval Foot and Ankle Bone, Interval Foot and Ankle SoftTissue, Interval Gastroc Soleus Lengthening, Interval Hams Lengthening,Interval Neural Rhizotomy, Interval Patellar Advance, Interval PsoasRelease, Interval Rectus Femoris Transfer, Interval Tibial DerotationOsteotomy

**Popliteal Angle (Unilateral)**: Age, Popliteal Angle (Unilateral), Initial Contact Pelvis Angle SagittalPlane, Initial Contact Knee Angle Sagittal Plane, Mean Stance Knee AngleSagittal Plane, Minimum Stance Knee Angle Sagittal Plane, GMFCS, IntervalAdductor Release, Interval DFEO + Patellar Advance, Interval FemoralDerotation Osteotomy, Interval Foot and Ankle Bone, Interval Foot andAnkle Soft Tissue, Interval Gastroc Soleus Lengthening, Interval HamsLengthening, Interval Neural Rhizotomy, Interval Patellar Advance, IntervalPsoas Release, Interval Rectus Femoris Transfer, Interval Tibial DerotationOsteotomy

**Mean Stance Foot Angle - Transverse Plane**: Age, EOS Femoral Anteversion, EOS Bimalleolar Axis Angle, TrochantericProminence Angle, Bimalleolar Axis Angle, Maximum External Hip Rotation,Maximum Internal Hip Rotation, Mean Stance Pelvis Angle Transverse Plane,Mean Stance Hip Angle Transverse Plane, Mean Stance Knee Angle TransversePlane, Mean Stance Foot Angle Transverse Plane, GMFCS, Interval AdductorRelease, Interval DFEO + Patellar Advance, Interval Femoral DerotationOsteotomy, Interval Foot and Ankle Bone, Interval Foot and Ankle SoftTissue, Interval Gastroc Soleus Lengthening, Interval Hams Lengthening,Interval Neural Rhizotomy, Interval Patellar Advance, Interval PsoasRelease, Interval Rectus Femoris Transfer, Interval Tibial DerotationOsteotomy

**Initial Contact Ankle Angle - Sagittal Plane**: Age, Maximum Ankle Dorsiflexion (Knee Extended), Maximum Ankle Dorsiflexion(Knee Flexed), Initial Contact Knee Angle Sagittal Plane, Initial ContactAnkle Angle Sagittal Plane, Mid-Stance Ankle Angle Sagittal Plane, MinimumStance Knee Angle Sagittal Plane, Minimum Swing Ankle Angle SagittalPlane, GMFCS, Interval Adductor Release, Interval DFEO + Patellar Advance,Interval Femoral Derotation Osteotomy, Interval Foot and Ankle Bone,Interval Foot and Ankle Soft Tissue, Interval Gastroc Soleus Lengthening,Interval Hams Lengthening, Interval Neural Rhizotomy, Interval PatellarAdvance, Interval Psoas Release, Interval Rectus Femoris Transfer, IntervalTibial Derotation Osteotomy

**Mean Stance Knee Angle - Transverse Plane**: Age, EOS Femoral Anteversion, EOS Bimalleolar Axis Angle, TrochantericProminence Angle, Bimalleolar Axis Angle, Maximum External Hip Rotation,Maximum Internal Hip Rotation, Mean Stance Pelvis Angle Transverse Plane,Mean Stance Hip Angle Transverse Plane, Mean Stance Knee Angle TransversePlane, Mean Stance Foot Angle Transverse Plane, GMFCS, Interval AdductorRelease, Interval DFEO + Patellar Advance, Interval Femoral DerotationOsteotomy, Interval Foot and Ankle Bone, Interval Foot and Ankle SoftTissue, Interval Gastroc Soleus Lengthening, Interval Hams Lengthening,Interval Neural Rhizotomy, Interval Patellar Advance, Interval PsoasRelease, Interval Rectus Femoris Transfer, Interval Tibial DerotationOsteotomy

**Max. Stance Hip Angle - Coronal Plane**: Age, Maximum Hip Abduction (Knee Extended), Initial Contact Hip AngleCoronal Plane, Maximum Stance Hip Angle Coronal Plane, Minimum Swing HipAngle Coronal Plane, GMFCS, Interval Adductor Release, Interval DFEO +Patellar Advance, Interval Femoral Derotation Osteotomy, Interval Foot andAnkle Bone, Interval Foot and Ankle Soft Tissue, Interval Gastroc SoleusLengthening, Interval Hams Lengthening, Interval Neural Rhizotomy, IntervalPatellar Advance, Interval Psoas Release, Interval Rectus Femoris Transfer,Interval Tibial Derotation Osteotomy

**Gait Deviation Index**: Overall Spasticity, Overall Weightbearing Foot Deformity, OverallNon-Weightbearing Foot Deformity, Age, EOS Femoral Anteversion, EOSBimalleolar Axis Angle, Maximum Ankle Dorsiflexion (Knee Extended),Maximum Ankle Dorsiflexion (Knee Flexed), Trochanteric Prominence Angle,Bimalleolar Axis Angle, Extensor Lag, Maximum External Hip Rotation,Maximum Internal Hip Rotation, Maximum Knee Extension, Patella Alta,Popliteal Angle (Unilateral), Gait Deviation Index, Maximum Hip Extension,Initial Contact Pelvis Angle Coronal Plane, Initial Contact Pelvis AngleSagittal Plane, Initial Contact Pelvis Angle Transverse Plane, InitialContact Hip Angle Coronal Plane, Initial Contact Hip Angle Sagittal Plane,Initial Contact Hip Angle Transverse Plane, Initial Contact Knee AngleSagittal Plane, Initial Contact Ankle Angle Sagittal Plane, Initial ContactFoot Angle Transverse Plane, Opposite Foot Off Pelvis Angle Coronal Plane,Opposite Foot Off Pelvis Angle Sagittal Plane, Opposite Foot Off PelvisAngle Transverse Plane, Opposite Foot Off Hip Angle Coronal Plane, OppositeFoot Off Hip Angle Sagittal Plane, Opposite Foot Off Hip Angle TransversePlane, Opposite Foot Off Knee Angle Sagittal Plane, Opposite Foot OffAnkle Angle Sagittal Plane, Opposite Foot Off Foot Angle Transverse Plane,Opposite Foot Contact Pelvis Angle Coronal Plane, Opposite Foot ContactPelvis Angle Sagittal Plane, Opposite Foot Contact Pelvis Angle TransversePlane, Opposite Foot Contact Hip Angle Coronal Plane, Opposite Foot ContactHip Angle Sagittal Plane, Opposite Foot Contact Hip Angle Transverse Plane,Opposite Foot Contact Knee Angle Sagittal Plane, Opposite Foot ContactAnkle Angle Sagittal Plane, Opposite Foot Contact Foot Angle TransversePlane, Foot Off Pelvis Angle Coronal Plane, Foot Off Pelvis Angle SagittalPlane, Foot Off Pelvis Angle Transverse Plane, Foot Off Hip Angle CoronalPlane, Foot Off Hip Angle Sagittal Plane, Foot Off Hip Angle TransversePlane, Foot Off Knee Angle Sagittal Plane, Foot Off Ankle Angle SagittalPlane, Foot Off Foot Angle Transverse Plane, Mid-Swing Pelvis AngleCoronal Plane, Mid-Swing Pelvis Angle Sagittal Plane, Mid-Swing PelvisAngle Transverse Plane, Mid-Swing Hip Angle Coronal Plane, Mid-Swing HipAngle Sagittal Plane, Mid-Swing Hip Angle Transverse Plane, Mid-Swing KneeAngle Sagittal Plane, Mid-Swing Ankle Angle Sagittal Plane, Mid-Swing FootAngle Transverse Plane, GMFCS, Interval Adductor Release, Interval DFEO +Patellar Advance, Interval Femoral Derotation Osteotomy, Interval Foot andAnkle Bone, Interval Foot and Ankle Soft Tissue, Interval Gastroc SoleusLengthening, Interval Hams Lengthening, Interval Neural Rhizotomy, IntervalPatellar Advance, Interval Psoas Release, Interval Rectus Femoris Transfer,Interval Tibial Derotation Osteotomy

**Functional Assessment Questionnaire Transform**: Overall Spasticity, Age, Functional Assessment Questionnaire Transform,GMFCS, Interval Adductor Release, Interval DFEO + Patellar Advance,Interval Femoral Derotation Osteotomy, Interval Foot and Ankle Bone,Interval Foot and Ankle Soft Tissue, Interval Gastroc Soleus Lengthening,Interval Hams Lengthening, Interval Neural Rhizotomy, Interval PatellarAdvance, Interval Psoas Release, Interval Rectus Femoris Transfer, IntervalTibial Derotation Osteotomy
