# Supplementary material for: Evidence Based Gait Analysis Interpretation Tools (EB-GAIT) treatment recommendation and outcome prediction models to support decision-making based on clinical gait analysis data
Source: PLoS One. 2025 Jul 29;20(7):e0328036. doi: 10.1371/journal.pone.0328036 (PMC12306754; doi:10.1371/journal.pone.0328036)
Supplement: S3 Sample_Report.html — (HTML) [file pone.0328036.s003.html]

 
  

 
 

 


 EB GAIT 
 


 
 
 
 
 
 
 
 
 
 
 
 
 

   
  
   


 

 

 

 
 
   
     Section 
   
   
    Arthur Dent 17199 Jun 2018  
    Background  
    Video  
    Treatment Assignment  
    Supporting Data  
    Other Conditions  
   
 
 
 
 
 

 
 
 EB GAIT 
 Evidence Based Gait Analysis Interpretation Technique 
 


 

    
  
    
   
  


 


 
 Arthur Dent 17199 Jun 2018 
 
 
 Background 
   
   Patient    Patient History    Gait Outcome Assessment List (GOAL)   
 
 
 
 
 
 
 
   
     
     
     
     
     
     
   
   
     
       Arthur Dent (17199), Exam Date: Jun 2018 
     
    
     
       Age 
       Diagnosis 
       Diagnosis Modifier 
       Diagnosis Side 
       GMFCS 
       Functional Assessment Questionnaire 
     
   
   
      8.9 
 Cerebral palsy 
 Hemiplegia 
 Right 
 II 
 09-Walks community distances; OK with curbs, uneven terrain; needs help on stairs, climbing  
   
  
  
 
 
 
 
 
 
   
   Birth    Gait    Treatment   
 
 
 
 
 
 
 
   
     
       Birth History 
     
     
       Gestation = 42 wks, Weight = ?lb ?oz, NICU = 0 wks, Ventilator = 0 wks 
     
   
   
     
       Prenatal 
     
      Unknown  
     
       Delivery 
     
      Lack of oxygen to the baby  
      Emergency c-section  
     
       Neonatal 
     
      Hyperbilirubinemia  
   
  
  
 
 
 
 
 
 
 
 
 
 
 
   
     
     
     
   
   
     
       Developmental History 
     
    
     
       Noticed Problem [mo] 
       First Step [yr] 
       Initial Walking Aid 
     
   
   
      3 
 2 
 Y  
   
  
  
 
 
 
 
 
 
 
 
 
 
 
   
     
     
     
     
     
   
   
     
       Treatment History 
     
    
     
       Treatment 
       Side 
       Description 
       Location 
       Type 
     
   
   
     
       2013, Age: 3 yr 
     
      Neural BOTOX 
 Right 
 motor point block 
 gastroc (lateral head)  
 botulinum toxin type A   
      Neural BOTOX 
 Right 
 motor point block 
 gastroc (medial head)  
 botulinum toxin type A   
     
       2013, Age: 4 yr 
     
      Neural BOTOX 
 Right 
 motor point block 
 gastroc (lateral head)  
 botulinum toxin type A   
      Neural BOTOX 
 Right 
 motor point block 
 gastroc (medial head)  
 botulinum toxin type A   
     
       2014, Age: 4 yr 
     
      Neural BOTOX 
 Right 
 motor point block 
 gastroc (lateral head)  
 botulinum toxin type A   
      Neural BOTOX 
 Right 
 motor point block 
 gastroc (medial head)  
 botulinum toxin type A   
     
       2014, Age: 5 yr 
     
      Neural BOTOX 
 Right 
 motor point block 
 gastroc (lateral head)  
 botulinum toxin type A   
      Neural BOTOX 
 Right 
 motor point block 
 gastroc (medial head)  
 botulinum toxin type A   
     
       2015, Age: 5 yr 
     
      Neural BOTOX 
 Right 
 motor point block 
 gastroc (lateral head)  
 botulinum toxin type A   
      Neural BOTOX 
 Right 
 motor point block 
 gastroc (medial head)  
 botulinum toxin type A   
     
       2016, Age: 6 yr 
     
      Neural BOTOX 
 Right 
 motor point block 
 gastroc (lateral head)  
 botulinum toxin type A   
      Neural BOTOX 
 Right 
 motor point block 
 gastroc (medial head)  
 botulinum toxin type A   
     
       2017, Age: 7 yr 
     
      Neural BOTOX 
 Right 
 motor point block 
 gastroc (lateral head)  
 botulinum toxin type A   
      Neural BOTOX 
 Right 
 motor point block 
 gastroc (medial head)  
 botulinum toxin type A   
     
       2017, Age: 8 yr 
     
      Neural BOTOX 
 Right 
 motor point block 
 gastroc (lateral head)  
 botulinum toxin type A   
      Neural BOTOX 
 Right 
 motor point block 
 gastroc (medial head)  
 botulinum toxin type A   
     
       2018, Age: 8 yr 
     
      Neural BOTOX 
 Right 
 motor point block 
 gastroc (lateral head)  
 botulinum toxin type A   
      Neural BOTOX 
 Right 
 motor point block 
 gastroc (medial head)  
 botulinum toxin type A   
   
  
  
 
 
 
 
 
 
 
 
 
   
   GOAL Summary    Very Important GOALs    GOAL History   
 
 
 
 
 
 
 
   
     
     
     
   
   
     
       GOAL Domain Scores 
     
    
     
       Domain 
       Score 
       Percentile 
     
   
   
      TOTAL Score 
 50 
 34%  
      Activities Sports Rec 
 27 
 55%  
      ADL Indep 
 59 
 28%  
      Braces Mobility 
 25 
 12%  
      Gait Func Mobility 
 77 
 90%  
      Gait Pattern Appearance 
 17 
 10%  
      Image Esteem 
 46 
 36%  
      Pain Discomfort Fatigue 
 63 
 29%  
   
   
     
          Severity  is based on percentile compared to GMFCS matched peers   
     
     
          Scores  are raw values out of 100   
     
   
  
 
 
 
 
 
 
 
 
 
 
 
   
     
     
   
   
     
       Very Important GOALs 
     
    
   
   
     
       Activities of Daily Living 
     
      Washing/bathing his/her self 
 Very Difficult  
      Getting dressed 
 Difficult  
     
       Gait Function and Mobility 
     
      Walking for more than 250 meters 
 Slightly Difficult  
      Walking for more than 15 minutes 
 Slightly Difficult  
      Walking faster than usual 
 Slightly Difficult  
      Going up and down stairs 
 Easy  
      Going up and down slopes 
 Slightly Difficult  
      Walking on uneven ground 
 Slightly Difficult  
      Walking on slippery surfaces 
 Difficult  
     
       Pain, Discomfort, and Fatigue 
     
      Pain or discomfort in the feet or ankles 
 A Few Times (once a week)  
      Pain or discomfort in the lower legs 
 Fairly Often (2 to 3 times a week)  
      Pain or discomfort in the knees 
 A Few Times (once a week)  
      Pain or discomfort in the thighs or hips 
 Once or Twice  
      Feeling tired while walking 
 Every Day  
      Feeling tired during any other physical activities 
 Fairly Often (2 to 3 times a week)  
     
       Physical Activities, Sports, and Rec. 
     
      Running 
 Difficult  
      Participating in activities that require balance 
 Slightly Difficult  
      Climbing 
 Extremely Difficult / Impossible  
     
       Gait Pattern and Appearance 
     
      Walking without tripping and falling 
 Slightly Difficult  
      Wearing footwear of my choice 
 Slightly Difficult  
     
       Braces and Mobility Aids 
     
      Wearing braces or orthotics 
 Unhappy  
     
       Body Image and Self-Esteem 
     
      The way you get around compared with others 
 Unhappy  
   
  
  
 
 
 
 
 
 
 
 
 
 
     
 
 
 
 
 
 
 
 
 
 
 
 
 Video 
   
  
 

 
 
 
 
    
       8yr_11mo 
      
    
     
       
         
        Your browser does not support the video tag.
       
        Full Body Run Orthoses  Full Body Walk Barefoot  Full Body Run Barefoot  Lower Leg Walk Barefoot - AP  Lower Leg Walk Barefoot - Sag  Feet  Root Sign  Functional Feet  Play All     

 
 
 
 Treatment Assignment 
   
   Propensity Profile    Clinical Reasoning    Outcome Details   
 
 
 The likelihood that the limb would have undergone the indicated surgery based on historical practice standards. 
 
 
  
 Meeting historical standards  does not  ensure a good outcome. 
Check  Outcome Predictions  tab for additional information. 
 
 
 
 
 
 
 
   
     
     
     
   
   
     
          Patient Propensity Profile    
     
    
     
       Surgery 
       Left 
       Right 
     
   
   
      Femoral Derotation Osteotomy 
 Somewhat Unlikely 
 Somewhat Likely  
      Tibial Derotation Osteotomy 
 Somewhat Unlikely 
 Neither  
      Psoas Release 
 Unlikely 
 Somewhat Unlikely  
      Hams Lengthening 
 Unlikely 
 Somewhat Likely  
      Adductor Release 
 Unlikely 
 Somewhat Unlikely  
      Gastroc Soleus Lengthening 
 Unlikely 
 Likely  
      DFEO Patellar Advance 
 Unlikely 
 Unlikely  
      Patellar Advance 
 Unlikely 
 Unlikely  
      Foot and Ankle Bone 
 Unlikely 
 Likely  
      Foot and Ankle Soft Tissue 
 Unlikely 
 Neither  
      Neural Rhizotomy 
 Unlikely 
 Somewhat Unlikely  
      Rectus Transfer 
 Unlikely 
 Unlikely  
   
   
     
       Unlikely [0%-20%), Somewhat Unlikely [20%-40%), Neither [40%-60%), Somewhat Likely [60%-80%), Likely [80%-100%) 
     
   
  
 
 
 
 
 
 
 Clinical factors underlying the historical practice standard, arranged from largest positive contributor (  indications  ) to largest negative contributor (  counter-indications  ). The top row shows the median (5%, 95%) surgery likelihood, color coded from   high likelihood   to   low likelihood  . The range in propensities accounts for measurement error variability in practice. The 🚶🏽‍♂️️ symbol indicates a kinematic variable    
   
   Femoral Derotation Osteotomy    Tibial Derotation Osteotomy    Psoas Release    Hams Lengthening    Adductor Release    Gastroc Soleus Lengthening    DFEO Patellar Advance    Patellar Advance    Foot and Ankle Bone    Foot and Ankle Soft Tissue    Neural Rhizotomy    Rectus Transfer   
 
 
 
 
 
 
 
 
 
 
 
 
 
 
 
Femoral Derotation Osteotomy
 
 
 
 
 
Left Propensity = 24% (8%, 44%)
 
 
 
blank
 
 
 
Right Propensity = 65% (47%, 81%)
 
 
 
 
 
Characteristic
 
 
Value
 
 
Characteristic
 
 
Value
 
 
 
 
 
 
Age
 
 
8.9
 
 
 
 
🚶🏽 Mean Stance Foot Angle Transverse Plane
 
 
-30
 
 
 
 
🚶🏽 Minimum Swing Knee Angle Coronal Plane
 
 
-14
 
 
 
 
Age
 
 
8.9
 
 
 
 
Prior Femoral Derotation Osteotomy
 
 
0
 
 
 
 
Femoral Torsion
 
 
35
 
 
 
 
Femoral Torsion
 
 
41
 
 
 
 
Prior Femoral Derotation Osteotomy
 
 
0
 
 
 
 
🚶🏽 Mean Stance Pelvis Angle Transverse Plane
 
 
11
 
 
 
 
🚶🏽 Mean Stance Hip Angle Transverse Plane
 
 
7
 
 
 
 
🚶🏽 Maximum Swing Knee Angle Coronal Plane
 
 
-4
 
 
 
 
🚶🏽 Minimum Swing Knee Angle Coronal Plane
 
 
-4
 
 
 
 
🚶🏽 Mean Stance Knee Angle Transverse Plane
 
 
-3
 
 
 
 
🚶🏽 Maximum Swing Knee Angle Coronal Plane
 
 
1
 
 
 
 
Bimalleolar Axis Angle
 
 
8
 
 
 
 
GMFCS
 
 
II
 
 
 
 
GMFCS
 
 
II
 
 
 
 
Affected Side
 
 
TRUE
 
 
 
 
🚶🏽 Mean Stance Foot Angle Transverse Plane
 
 
-3
 
 
 
 
Bimalleolar Axis Angle
 
 
15
 
 
 
 
Diagnosis
 
 
Cerebral palsy
 
 
 
 
🚶🏽 Mean Stance Pelvis Angle Transverse Plane
 
 
-10
 
 
 
 
Era
 
 
>2015
 
 
 
 
🚶🏽 Mean Stance Knee Angle Transverse Plane
 
 
-23
 
 
 
 
Maximum Internal Hip Rotation
 
 
50
 
 
 
 
Diagnosis
 
 
Cerebral palsy
 
 
 
 
Maximum External Hip Rotation
 
 
55
 
 
 
 
Era
 
 
>2015
 
 
 
 
Affected Side
 
 
FALSE
 
 
 
 
Maximum Internal Hip Rotation
 
 
54
 
 
 
 
🚶🏽 Mean Stance Hip Angle Transverse Plane
 
 
-11
 
 
 
 
Maximum External Hip Rotation
 
 
45
 
 
 
 
 
 
 
 
 
 
 
 
 
 
 
 
 
 
 
 
Tibial Derotation Osteotomy
 
 
 
 
 
Left Propensity = 28% (13%, 45%)
 
 
 
blank
 
 
 
Right Propensity = 42% (24%, 63%)
 
 
 
 
 
Characteristic
 
 
Value
 
 
Characteristic
 
 
Value
 
 
 
 
 
 
Age
 
 
8.9
 
 
 
 
Age
 
 
8.9
 
 
 
 
Femoral Torsion
 
 
41
 
 
 
 
🚶🏽 Mean Stance Foot Angle Transverse Plane
 
 
-30
 
 
 
 
GMFCS
 
 
II
 
 
 
 
🚶🏽 Mean Stance Knee Angle Transverse Plane
 
 
-23
 
 
 
 
🚶🏽 Mean Stance Pelvis Angle Transverse Plane
 
 
11
 
 
 
 
GMFCS
 
 
II
 
 
 
 
Prior Tibial Derotation Osteotomy
 
 
0
 
 
 
 
Prior Tibial Derotation Osteotomy
 
 
0
 
 
 
 
🚶🏽 Mean Stance Hip Angle Transverse Plane
 
 
-11
 
 
 
 
Femoral Torsion
 
 
35
 
 
 
 
Maximum Internal Hip Rotation
 
 
50
 
 
 
 
Era
 
 
>2015
 
 
 
 
🚶🏽 Minimum Swing Knee Angle Coronal Plane
 
 
-14
 
 
 
 
Affected Side
 
 
TRUE
 
 
 
 
Era
 
 
>2015
 
 
 
 
Maximum Internal Hip Rotation
 
 
54
 
 
 
 
Maximum External Hip Rotation
 
 
55
 
 
 
 
🚶🏽 Mean Stance Hip Angle Transverse Plane
 
 
7
 
 
 
 
🚶🏽 Maximum Swing Knee Angle Coronal Plane
 
 
-4
 
 
 
 
🚶🏽 Minimum Swing Knee Angle Coronal Plane
 
 
-4
 
 
 
 
Diagnosis
 
 
Cerebral palsy
 
 
 
 
🚶🏽 Mean Stance Pelvis Angle Transverse Plane
 
 
-10
 
 
 
 
🚶🏽 Mean Stance Knee Angle Transverse Plane
 
 
-3
 
 
 
 
Maximum External Hip Rotation
 
 
45
 
 
 
 
🚶🏽 Mean Stance Foot Angle Transverse Plane
 
 
-3
 
 
 
 
🚶🏽 Maximum Swing Knee Angle Coronal Plane
 
 
1
 
 
 
 
Affected Side
 
 
FALSE
 
 
 
 
Diagnosis
 
 
Cerebral palsy
 
 
 
 
Bimalleolar Axis Angle
 
 
8
 
 
 
 
Bimalleolar Axis Angle
 
 
15
 
 
 
 
 
 
 
 
 
 
 
 
 
 
 
 
 
 
 
 
Psoas Release
 
 
 
 
 
Left Propensity = 9% (1%, 27%)
 
 
 
blank
 
 
 
Right Propensity = 38% (16%, 63%)
 
 
 
 
 
Characteristic
 
 
Value
 
 
Characteristic
 
 
Value
 
 
 
 
 
 
Age
 
 
8.9
 
 
 
 
Age
 
 
8.9
 
 
 
 
GMFCS
 
 
II
 
 
 
 
🚶🏽 Minimum Stance Hip Angle Sagittal Plane
 
 
5
 
 
 
 
🚶🏽 Maximum Stance Pelvis Angle Sagittal Plane
 
 
21
 
 
 
 
🚶🏽 Maximum Stance Pelvis Angle Sagittal Plane
 
 
22
 
 
 
 
🚶🏽 Mean Stance Pelvis Angle Sagittal Plane
 
 
17
 
 
 
 
GMFCS
 
 
II
 
 
 
 
Popliteal Angle (Unilateral)
 
 
48
 
 
 
 
Popliteal Angle (Unilateral)
 
 
65
 
 
 
 
Diagnosis
 
 
Cerebral palsy
 
 
 
 
Affected Side
 
 
TRUE
 
 
 
 
Affected Side
 
 
FALSE
 
 
 
 
Diagnosis
 
 
Cerebral palsy
 
 
 
 
🚶🏽 Minimum Stance Hip Angle Sagittal Plane
 
 
-2
 
 
 
 
🚶🏽 Mean Stance Pelvis Angle Sagittal Plane
 
 
18
 
 
 
 
🚶🏽 Minimum Stance Pelvis Angle Sagittal Plane
 
 
16
 
 
 
 
🚶🏽 Minimum Stance Pelvis Angle Sagittal Plane
 
 
15
 
 
 
 
Era
 
 
>2015
 
 
 
 
Era
 
 
>2015
 
 
 
 
Maximum Hip Extension
 
 
0
 
 
 
 
Maximum Hip Extension
 
 
0
 
 
 
 
 
 
 
 
 
 
 
 
 
 
 
 
 
 
 
 
Hams Lengthening
 
 
 
 
 
Left Propensity = 1% (0%, 6%)
 
 
 
blank
 
 
 
Right Propensity = 61% (39%, 80%)
 
 
 
 
 
Characteristic
 
 
Value
 
 
Characteristic
 
 
Value
 
 
 
 
 
 
🚶🏽 Initial Contact Hip Angle Sagittal Plane
 
 
34
 
 
 
 
🚶🏽 Initial Contact Hip Angle Sagittal Plane
 
 
39
 
 
 
 
Age
 
 
8.9
 
 
 
 
Popliteal Angle (Unilateral)
 
 
65
 
 
 
 
Prior Hams Lengthening
 
 
0
 
 
 
 
Age
 
 
8.9
 
 
 
 
GMFCS
 
 
II
 
 
 
 
🚶🏽 Maximum Swing Semimembranosus Length
 
 
1.01
 
 
 
 
Diagnosis
 
 
Cerebral palsy
 
 
 
 
🚶🏽 Initial Contact Pelvis Angle Sagittal Plane
 
 
15
 
 
 
 
Maximum Knee Extension
 
 
-4
 
 
 
 
Affected Side
 
 
TRUE
 
 
 
 
🚶🏽 Initial Contact Pelvis Angle Sagittal Plane
 
 
21
 
 
 
 
🚶🏽 Initial Contact Knee Angle Sagittal Plane
 
 
22
 
 
 
 
Popliteal Angle (Unilateral)
 
 
48
 
 
 
 
Prior Hams Lengthening
 
 
0
 
 
 
 
🚶🏽 Minimum Stance Knee Angle Sagittal Plane
 
 
-1
 
 
 
 
GMFCS
 
 
II
 
 
 
 
Era
 
 
>2015
 
 
 
 
Diagnosis
 
 
Cerebral palsy
 
 
 
 
🚶🏽 Maximum Swing Semimembranosus Length
 
 
1.05
 
 
 
 
Maximum Knee Extension
 
 
0
 
 
 
 
Affected Side
 
 
FALSE
 
 
 
 
🚶🏽 Minimum Stance Knee Angle Sagittal Plane
 
 
8
 
 
 
 
🚶🏽 Initial Contact Knee Angle Sagittal Plane
 
 
-1
 
 
 
 
Era
 
 
>2015
 
 
 
 
 
 
 
 
 
 
 
 
 
 
 
 
 
 
 
 
Adductor Release
 
 
 
 
 
Left Propensity = 11% (2%, 24%)
 
 
 
blank
 
 
 
Right Propensity = 25% (9%, 47%)
 
 
 
 
 
Characteristic
 
 
Value
 
 
Characteristic
 
 
Value
 
 
 
 
 
 
Age
 
 
8.9
 
 
 
 
🚶🏽 Initial Contact Hip Angle Coronal Plane
 
 
6
 
 
 
 
Affected Side
 
 
FALSE
 
 
 
 
Age
 
 
8.9
 
 
 
 
Diagnosis
 
 
Cerebral palsy
 
 
 
 
Maximum Hip Abduction (Knee Flexed)
 
 
57
 
 
 
 
🚶🏽 Maximum Stance Hip Angle Coronal Plane
 
 
4
 
 
 
 
Hip Abductor Strength
 
 
3
 
 
 
 
🚶🏽 Minimum Swing Hip Angle Coronal Plane
 
 
-5
 
 
 
 
🚶🏽 Minimum Swing Hip Angle Coronal Plane
 
 
-3
 
 
 
 
Prior Adductor Release
 
 
0
 
 
 
 
Diagnosis
 
 
Cerebral palsy
 
 
 
 
GMFCS
 
 
II
 
 
 
 
GMFCS
 
 
II
 
 
 
 
Maximum Hip Abduction (Knee Flexed)
 
 
70
 
 
 
 
Affected Side
 
 
TRUE
 
 
 
 
🚶🏽 Initial Contact Hip Angle Coronal Plane
 
 
-9
 
 
 
 
Prior Adductor Release
 
 
0
 
 
 
 
Hip Abductor Strength
 
 
4
 
 
 
 
🚶🏽 Maximum Stance Hip Angle Coronal Plane
 
 
12
 
 
 
 
Maximum Hip Abduction (Knee Extended)
 
 
36
 
 
 
 
Maximum Hip Abduction (Knee Extended)
 
 
38
 
 
 
 
Era
 
 
>2015
 
 
 
 
Era
 
 
>2015
 
 
 
 
 
 
 
 
 
 
 
 
 
 
 
 
 
 
 
 
Gastroc Soleus Lengthening
 
 
 
 
 
Left Propensity = 8% (3%, 17%)
 
 
 
blank
 
 
 
Right Propensity = 81% (68%, 91%)
 
 
 
 
 
Characteristic
 
 
Value
 
 
Characteristic
 
 
Value
 
 
 
 
 
 
Age
 
 
8.9
 
 
 
 
🚶🏽 Initial Contact Ankle Angle Sagittal Plane
 
 
-13
 
 
 
 
Prior Gastroc Soleus Lengthening
 
 
0
 
 
 
 
Age
 
 
8.9
 
 
 
 
Maximum Ankle Dorsiflexion (Knee Extended)
 
 
4
 
 
 
 
Maximum Ankle Dorsiflexion (Knee Flexed)
 
 
0
 
 
 
 
Plantarflexor Strength
 
 
2
 
 
 
 
Maximum Ankle Dorsiflexion (Knee Extended)
 
 
-10
 
 
 
 
🚶🏽 Initial Contact Knee Angle Sagittal Plane
 
 
-1
 
 
 
 
Prior Gastroc Soleus Lengthening
 
 
0
 
 
 
 
Diagnosis
 
 
Cerebral palsy
 
 
 
 
Weightbearing Foot Position Severity
 
 
MOD
 
 
 
 
GMFCS
 
 
II
 
 
 
 
Weightbearing Foot Position
 
 
VAL
 
 
 
 
🚶🏽 Initial Contact Ankle Angle Sagittal Plane
 
 
-3
 
 
 
 
🚶🏽 Initial Contact Knee Angle Sagittal Plane
 
 
22
 
 
 
 
Plantarflexor Spasticity
 
 
0
 
 
 
 
Plantarflexor Spasticity
 
 
1
 
 
 
 
Weightbearing Foot Position
 
 
TYP
 
 
 
 
Affected Side
 
 
TRUE
 
 
 
 
Weightbearing Foot Position Severity
 
 
NONE
 
 
 
 
GMFCS
 
 
II
 
 
 
 
Maximum Ankle Dorsiflexion (Knee Flexed)
 
 
22
 
 
 
 
Plantarflexor Strength
 
 
2
 
 
 
 
Affected Side
 
 
FALSE
 
 
 
 
Diagnosis
 
 
Cerebral palsy
 
 
 
 
Era
 
 
>2015
 
 
 
 
Era
 
 
>2015
 
 
 
 
 
 
 
 
 
 
 
 
 
 
 
 
 
 
 
 
DFEO Patellar Advance
 
 
 
 
 
Left Propensity = 1% (0%, 2%)
 
 
 
blank
 
 
 
Right Propensity = 0% (0%, 1%)
 
 
 
 
 
Characteristic
 
 
Value
 
 
Characteristic
 
 
Value
 
 
 
 
 
 
Affected Side
 
 
FALSE
 
 
 
 
Prior Patellar Advance
 
 
0
 
 
 
 
Prior Patellar Advance
 
 
0
 
 
 
 
Prior DFEO + Patellar Advance
 
 
0
 
 
 
 
Prior DFEO + Patellar Advance
 
 
0
 
 
 
 
Prior DFEO
 
 
0
 
 
 
 
Prior DFEO
 
 
0
 
 
 
 
Diagnosis
 
 
Cerebral palsy
 
 
 
 
Diagnosis
 
 
Cerebral palsy
 
 
 
 
Affected Side
 
 
TRUE
 
 
 
 
Era
 
 
>2015
 
 
 
 
Era
 
 
>2015
 
 
 
 
Prior Hams Lengthening
 
 
0
 
 
 
 
Prior Hams Lengthening
 
 
0
 
 
 
 
GMFCS
 
 
II
 
 
 
 
Extensor Lag
 
 
0
 
 
 
 
Extensor Lag
 
 
0
 
 
 
 
GMFCS
 
 
II
 
 
 
 
Patella Alta
 
 
N
 
 
 
 
Patella Alta
 
 
N
 
 
 
 
Age
 
 
8.9
 
 
 
 
Maximum Knee Extension
 
 
0
 
 
 
 
Maximum Knee Extension
 
 
-4
 
 
 
 
Age
 
 
8.9
 
 
 
 
🚶🏽 Mid-Stance Knee Angle Sagittal Plane
 
 
9
 
 
 
 
🚶🏽 Mid-Stance Knee Angle Sagittal Plane
 
 
11
 
 
 
 
 
 
 
 
 
 
 
 
 
 
 
 
 
 
 
 
Patellar Advance
 
 
 
 
 
Left Propensity = 7% (0%, 23%)
 
 
 
blank
 
 
 
Right Propensity = 7% (1%, 20%)
 
 
 
 
 
Characteristic
 
 
Value
 
 
Characteristic
 
 
Value
 
 
 
 
 
 
Era
 
 
>2015
 
 
 
 
Era
 
 
>2015
 
 
 
 
GMFCS
 
 
II
 
 
 
 
GMFCS
 
 
II
 
 
 
 
Affected Side
 
 
FALSE
 
 
 
 
Maximum Knee Extension
 
 
0
 
 
 
 
Age
 
 
8.9
 
 
 
 
Prior Patellar Advance
 
 
0
 
 
 
 
Prior Patellar Advance
 
 
0
 
 
 
 
Prior DFEO + Patellar Advance
 
 
0
 
 
 
 
Prior DFEO + Patellar Advance
 
 
0
 
 
 
 
Prior DFEO
 
 
0
 
 
 
 
Prior DFEO
 
 
0
 
 
 
 
Affected Side
 
 
TRUE
 
 
 
 
Diagnosis
 
 
Cerebral palsy
 
 
 
 
Diagnosis
 
 
Cerebral palsy
 
 
 
 
Maximum Knee Extension
 
 
-4
 
 
 
 
Prior Hams Lengthening
 
 
0
 
 
 
 
Prior Hams Lengthening
 
 
0
 
 
 
 
🚶🏽 Mid-Stance Knee Angle Sagittal Plane
 
 
11
 
 
 
 
🚶🏽 Mid-Stance Knee Angle Sagittal Plane
 
 
9
 
 
 
 
Age
 
 
8.9
 
 
 
 
Extensor Lag
 
 
0
 
 
 
 
Extensor Lag
 
 
0
 
 
 
 
Patella Alta
 
 
N
 
 
 
 
Patella Alta
 
 
N
 
 
 
 
 
 
 
 
 
 
 
 
 
 
 
 
 
 
 
 
Foot and Ankle Bone
 
 
 
 
 
Left Propensity = 5% (1%, 10%)
 
 
 
blank
 
 
 
Right Propensity = 89% (80%, 96%)
 
 
 
 
 
Characteristic
 
 
Value
 
 
Characteristic
 
 
Value
 
 
 
 
 
 
Non-Weightbearing Midfoot Motion
 
 
HYP
 
 
 
 
🚶🏽 Mean Stance Foot Angle Transverse Plane
 
 
-30
 
 
 
 
Age
 
 
8.9
 
 
 
 
Weightbearing Midfoot Position
 
 
PLA
 
 
 
 
Prior Foot and Ankle Bone
 
 
0
 
 
 
 
Weightbearing Forefoot Ab/Adduction
 
 
ABD
 
 
 
 
GMFCS
 
 
II
 
 
 
 
Weightbearing Foot Position
 
 
VAL
 
 
 
 
Diagnosis
 
 
Cerebral palsy
 
 
 
 
Weightbearing Forefoot Varus/Valgus Severity
 
 
MIL
 
 
 
 
Weightbearing Forefoot Varus/Valgus
 
 
TYP
 
 
 
 
Non-Weightbearing Midfoot Motion
 
 
HYP
 
 
 
 
Prior Foot and Ankle Soft Tissue
 
 
0
 
 
 
 
Weightbearing Foot Position Severity
 
 
MOD
 
 
 
 
Weightbearing Forefoot Ab/Adduction Severity
 
 
NONE
 
 
 
 
Age
 
 
8.9
 
 
 
 
Weightbearing Midfoot Position
 
 
TYP
 
 
 
 
Weightbearing Forefoot Ab/Adduction Severity
 
 
MOD
 
 
 
 
Non-Weightbearing Arch
 
 
TYP
 
 
 
 
Prior Foot and Ankle Bone
 
 
0
 
 
 
 
Weightbearing Forefoot Varus/Valgus Severity
 
 
NONE
 
 
 
 
Weightbearing Forefoot Varus/Valgus
 
 
VAL
 
 
 
 
🚶🏽 Mean Stance Foot Angle Transverse Plane
 
 
-3
 
 
 
 
Affected Side
 
 
TRUE
 
 
 
 
Era
 
 
>2015
 
 
 
 
GMFCS
 
 
II
 
 
 
 
Weightbearing Forefoot Ab/Adduction
 
 
TYP
 
 
 
 
Prior Foot and Ankle Soft Tissue
 
 
0
 
 
 
 
Weightbearing Foot Position Severity
 
 
NONE
 
 
 
 
Diagnosis
 
 
Cerebral palsy
 
 
 
 
Affected Side
 
 
FALSE
 
 
 
 
Non-Weightbearing Arch
 
 
TYP
 
 
 
 
Weightbearing Foot Position
 
 
TYP
 
 
 
 
Era
 
 
>2015
 
 
 
 
 
 
 
 
 
 
 
 
 
 
 
 
 
 
 
 
Foot and Ankle Soft Tissue
 
 
 
 
 
Left Propensity = 5% (1%, 12%)
 
 
 
blank
 
 
 
Right Propensity = 46% (25%, 68%)
 
 
 
 
 
Characteristic
 
 
Value
 
 
Characteristic
 
 
Value
 
 
 
 
 
 
Age
 
 
8.9
 
 
 
 
Weightbearing Forefoot Ab/Adduction Severity
 
 
MOD
 
 
 
 
Prior Foot and Ankle Bone
 
 
0
 
 
 
 
Weightbearing Forefoot Varus/Valgus
 
 
VAL
 
 
 
 
GMFCS
 
 
II
 
 
 
 
Weightbearing Forefoot Ab/Adduction
 
 
ABD
 
 
 
 
Prior Foot and Ankle Soft Tissue
 
 
0
 
 
 
 
🚶🏽 Mean Swing Foot Angle Transverse Plane
 
 
-27
 
 
 
 
Non-Weightbearing Hindfoot Varus/Valgus Severity
 
 
NONE
 
 
 
 
Age
 
 
8.9
 
 
 
 
First Ray Plantarflexion
 
 
TYP
 
 
 
 
🚶🏽 Mean Stance Foot Angle Transverse Plane
 
 
-30
 
 
 
 
Weightbearing Midfoot Position
 
 
TYP
 
 
 
 
GMFCS
 
 
II
 
 
 
 
🚶🏽 Mean Stance Foot Angle Transverse Plane
 
 
-3
 
 
 
 
Weightbearing Foot Position Severity
 
 
MOD
 
 
 
 
Non-Weightbearing Hindfoot Varus/Valgus
 
 
VER
 
 
 
 
Weightbearing Foot Position
 
 
VAL
 
 
 
 
Weightbearing Forefoot Varus/Valgus Severity
 
 
NONE
 
 
 
 
Weightbearing Forefoot Varus/Valgus Severity
 
 
MIL
 
 
 
 
Diagnosis
 
 
Cerebral palsy
 
 
 
 
Prior Foot and Ankle Bone
 
 
0
 
 
 
 
🚶🏽 Mean Swing Foot Angle Transverse Plane
 
 
0
 
 
 
 
Affected Side
 
 
TRUE
 
 
 
 
Non-Weightbearing Midfoot Motion
 
 
HYP
 
 
 
 
Non-Weightbearing Hindfoot Varus/Valgus Severity
 
 
NONE
 
 
 
 
Non-Weightbearing Forefoot Varus/Valgus Severity
 
 
NONE
 
 
 
 
Prior Foot and Ankle Soft Tissue
 
 
0
 
 
 
 
Weightbearing Forefoot Ab/Adduction Severity
 
 
NONE
 
 
 
 
Non-Weightbearing Hindfoot Varus/Valgus
 
 
VER
 
 
 
 
Non-Weightbearing Forefoot Varus/Valgus
 
 
NEU
 
 
 
 
First Ray Plantarflexion
 
 
TYP
 
 
 
 
Weightbearing Foot Position
 
 
TYP
 
 
 
 
Non-Weightbearing Midfoot Motion
 
 
HYP
 
 
 
 
Weightbearing Foot Position Severity
 
 
NONE
 
 
 
 
Diagnosis
 
 
Cerebral palsy
 
 
 
 
Non-Weightbearing Arch
 
 
TYP
 
 
 
 
Weightbearing Midfoot Position
 
 
PLA
 
 
 
 
Weightbearing Forefoot Varus/Valgus
 
 
TYP
 
 
 
 
Non-Weightbearing Forefoot Varus/Valgus Severity
 
 
NONE
 
 
 
 
Weightbearing Forefoot Ab/Adduction
 
 
TYP
 
 
 
 
Non-Weightbearing Forefoot Varus/Valgus
 
 
NEU
 
 
 
 
Affected Side
 
 
FALSE
 
 
 
 
Non-Weightbearing Arch
 
 
TYP
 
 
 
 
Era
 
 
>2015
 
 
 
 
Era
 
 
>2015
 
 
 
 
 
 
 
 
 
 
 
 
 
 
 
 
 
 
 
 
Neural Rhizotomy
 
 
 
 
 
Left Propensity = 9% (1%, 24%)
 
 
 
blank
 
 
 
Right Propensity = 34% (16%, 55%)
 
 
 
 
 
Characteristic
 
 
Value
 
 
Characteristic
 
 
Value
 
 
 
 
 
 
Era
 
 
>2015
 
 
 
 
Prior Neural Rhizotomy
 
 
0
 
 
 
 
Prior Neural Rhizotomy
 
 
0
 
 
 
 
Era
 
 
>2015
 
 
 
 
Dynamic Motor Control (Walking)
 
 
92
 
 
 
 
Plantarflexor Spasticity
 
 
1
 
 
 
 
Diagnosis
 
 
Cerebral palsy
 
 
 
 
Dynamic Motor Control (Walking)
 
 
92
 
 
 
 
🚶🏽 Maximum Stance Ankle Angle Sagittal Plane
 
 
15
 
 
 
 
🚶🏽 Mean Swing Ankle Angle Sagittal Plane
 
 
-18
 
 
 
 
🚶🏽 Initial Contact Ankle Angle Sagittal Plane
 
 
-3
 
 
 
 
🚶🏽 Maximum Stance Ankle Angle Sagittal Plane
 
 
1
 
 
 
 
Hamstring Spasticity
 
 
0
 
 
 
 
Hamstring Spasticity
 
 
1
 
 
 
 
Age
 
 
8.9
 
 
 
 
Diagnosis
 
 
Cerebral palsy
 
 
 
 
GMFCS
 
 
II
 
 
 
 
Affected Side
 
 
TRUE
 
 
 
 
🚶🏽 Mean Swing Ankle Angle Sagittal Plane
 
 
-1
 
 
 
 
Age
 
 
8.9
 
 
 
 
Rectus Femoris Spasticity
 
 
0
 
 
 
 
GMFCS
 
 
II
 
 
 
 
Affected Side
 
 
FALSE
 
 
 
 
🚶🏽 Initial Contact Ankle Angle Sagittal Plane
 
 
-13
 
 
 
 
🚶🏽 Initial Contact Knee Angle Sagittal Plane
 
 
-1
 
 
 
 
Rectus Femoris Spasticity
 
 
0
 
 
 
 
Net Dimensionless Oxygen Consumption (% Typ)
 
 
0.57
 
 
 
 
🚶🏽 Initial Contact Knee Angle Sagittal Plane
 
 
22
 
 
 
 
Adductor Spasticity
 
 
0
 
 
 
 
Adductor Spasticity
 
 
0
 
 
 
 
Plantarflexor Spasticity
 
 
0
 
 
 
 
Net Dimensionless Oxygen Consumption (% Typ)
 
 
0.57
 
 
 
 
 
 
 
 
 
 
 
 
 
 
 
 
 
 
 
 
Rectus Transfer
 
 
 
 
 
Left Propensity = 1% (0%, 3%)
 
 
 
blank
 
 
 
Right Propensity = 15% (2%, 38%)
 
 
 
 
 
Characteristic
 
 
Value
 
 
Characteristic
 
 
Value
 
 
 
 
 
 
Age
 
 
8.9
 
 
 
 
🚶🏽 ROMSwing Knee Angle Sagittal Plane
 
 
31
 
 
 
 
GMFCS
 
 
II
 
 
 
 
GMFCS
 
 
II
 
 
 
 
Prior Rectus Femoris Transfer
 
 
0
 
 
 
 
Age
 
 
8.9
 
 
 
 
🚶🏽 Maximum Swing Knee Angle Sagittal Plane
 
 
56
 
 
 
 
Prior Rectus Femoris Transfer
 
 
0
 
 
 
 
Prior Neural Rhizotomy
 
 
0
 
 
 
 
🚶🏽 Maximum Swing Knee Angle Sagittal Plane
 
 
57
 
 
 
 
🚶🏽 Foot Off Knee Angle Sagittal Plane
 
 
33
 
 
 
 
🚶🏽 Foot Off Knee Angle Sagittal Plane
 
 
25
 
 
 
 
Diagnosis
 
 
Cerebral palsy
 
 
 
 
Prior Neural Rhizotomy
 
 
0
 
 
 
 
Affected Side
 
 
FALSE
 
 
 
 
Affected Side
 
 
TRUE
 
 
 
 
Rectus Femoris Spasticity
 
 
0
 
 
 
 
Diagnosis
 
 
Cerebral palsy
 
 
 
 
🚶🏽 Initial Contact Knee Angle Sagittal Plane
 
 
-1
 
 
 
 
🚶🏽 Initial Contact Knee Angle Sagittal Plane
 
 
22
 
 
 
 
🚶🏽 ROMSwing Knee Angle Sagittal Plane
 
 
53
 
 
 
 
Rectus Femoris Spasticity
 
 
0
 
 
 
 
Era
 
 
>2015
 
 
 
 
Era
 
 
>2015
 
 
 
 
 
 
 
 
 
 
 Plots show   Change Scores  .   Treated   limbs underwent the surgery while   Control   limbs did not. The mean (point), 50% prediction interval (thick line), and 90% prediction interval (thin line) are shown.  
   
   Femoral Derotation Osteotomy    Tibial Derotation Osteotomy    Psoas Release    Hams Lengthening    Adductor Release    Gastroc Soleus Lengthening    DFEO Patellar Advance    Patellar Advance    Foot and Ankle Bone    Foot and Ankle Soft Tissue    Neural Rhizotomy    Rectus Transfer   
 
 
     
 
 
     
 
 
     
 
 
     
 
 
     
 
 
     
 
 
     
 
 
     
 
 
     
 
 
     
 
 
     
 
 
     
 
 
 
 
 
 
 
 
 Supporting Data 
   
   Physical Examination    Kinematics    Development   
 
 
 Cells are colored according to severity (  none  ,   mild  ,   moderate  ,   severe  ,   not applicable  ). 
   
   Torsion    Contracture    Motor Control & Strength    Spasticity    Weight-Bearing Foot    Non-Weight-Bearing Foot   
 
 
 
 
 
 
 
 
 
 
 
 
 
Measure
 
 
Left
 
 
Right
 
 
 
 
 
 
Femur
 
 
 
 
Maximum Internal Hip Rotation
 
 
50
 
 
54
 
 
 
 
Maximum External Hip Rotation
 
 
55
 
 
45
 
 
 
 
Trochanteric Prominence Angle
 
 
41
 
 
35
 
 
 
 
EOS Femoral Anteversion
 
 
—
 
 
—
 
 
 
 
Tibia
 
 
 
 
Bimalleolar Axis Angle
 
 
8
 
 
15
 
 
 
 
Second Toe Test
 
 
0
 
 
12
 
 
 
 
EOS Bimalleolar Axis Angle
 
 
—
 
 
—
 
 
 
 
 
 
 
 
 
 
 
 
 
 
 
 
 
 
Measure
 
 
Left
 
 
Right
 
 
 
 
 
 
Hip
 
 
 
 
Maximum Hip Flexion
 
 
109
 
 
123
 
 
 
 
Maximum Hip Extension
 
 
0
 
 
0
 
 
 
 
Maximum Hip Abduction (Knee Extended)
 
 
36
 
 
38
 
 
 
 
Maximum Hip Abduction (Knee Flexed)
 
 
70
 
 
57
 
 
 
 
Knee
 
 
 
 
Popliteal Angle (Unilateral)
 
 
48
 
 
65
 
 
 
 
Popliteal Angle (Bilateral)
 
 
38
 
 
53
 
 
 
 
Maximum Knee Extension
 
 
-4
 
 
0
 
 
 
 
Maximum Knee Flexion
 
 
144
 
 
148
 
 
 
 
Extensor Lag
 
 
0
 
 
0
 
 
 
 
Patella Alta
 
 
No
 
 
No
 
 
 
 
Ankle
 
 
 
 
Maximum Ankle Dorsiflexion (Knee Extended)
 
 
4
 
 
-10
 
 
 
 
Maximum Ankle Dorsiflexion (Knee Flexed)
 
 
22
 
 
0
 
 
 
 
 
 
 
 
 
 
 
 
 
 
 
 
 
 
 
 
 
Measure
 
 
 
Motor Control
 
 
 
blank
 
 
 
Strength
 
 
 
 
 
Left
 
 
Right
 
 
Left
 
 
Right
 
 
 
 
 
 
Core
 
 
 
 
Abdominal
 
 
2
 
 
2
 
 
 
 
4
 
 
4
 
 
 
 
Back Extensor
 
 
—
 
 
—
 
 
 
 
—
 
 
—
 
 
 
 
Hip
 
 
 
 
Hip Abductor
 
 
1
 
 
1
 
 
 
 
4
 
 
3
 
 
 
 
Hip Adductor
 
 
1
 
 
1
 
 
 
 
4
 
 
—
 
 
 
 
Hip Extensor (Knee Flexed)
 
 
1
 
 
—
 
 
 
 
—
 
 
—
 
 
 
 
Hip Extensor
 
 
1
 
 
—
 
 
 
 
—
 
 
—
 
 
 
 
Hip Flexor
 
 
1
 
 
1
 
 
 
 
5
 
 
5
 
 
 
 
Knee
 
 
 
 
Knee Extensor
 
 
2
 
 
1
 
 
 
 
5
 
 
4
 
 
 
 
Knee Flexor
 
 
2
 
 
1
 
 
 
 
5
 
 
4
 
 
 
 
Ankle/Foot
 
 
 
 
Anterior Tibialis
 
 
2
 
 
0
 
 
 
 
5
 
 
2
 
 
 
 
Extensor Hallucis Longus
 
 
2
 
 
0
 
 
 
 
5
 
 
0
 
 
 
 
Flexor Hallucis Longus
 
 
2
 
 
0
 
 
 
 
5
 
 
0
 
 
 
 
Peroneus Brevis
 
 
2
 
 
0
 
 
 
 
5
 
 
0
 
 
 
 
Peroneus Longus
 
 
2
 
 
0
 
 
 
 
5
 
 
0
 
 
 
 
Plantarflexor
 
 
1
 
 
0
 
 
 
 
2
 
 
2
 
 
 
 
Posterior Tibialis
 
 
2
 
 
0
 
 
 
 
5
 
 
0
 
 
 
 
 
 
 
 
 
 
 
 
 
 
 
 
 
 
Measure
 
 
Left
 
 
Right
 
 
 
 
 
 
Hip Flexor Spasticity
 
 
0
 
 
0
 
 
 
 
Adductor Spasticity
 
 
0
 
 
0
 
 
 
 
Hamstring Spasticity
 
 
0
 
 
1
 
 
 
 
Rectus Femoris Spasticity
 
 
0
 
 
0
 
 
 
 
Plantarflexor Spasticity
 
 
0
 
 
1
 
 
 
 
Posterior Tibialis Spasticity
 
 
0
 
 
0
 
 
 
 
Ankle Clonus
 
 
—
 
 
—
 
 
 
 
 
 
 
 
 
 
 
 
 
 
 
 
 
 
Measure
 
 
Left
 
 
Right
 
 
 
 
 
 
Weightbearing Forefoot Varus/Valgus
 
 
TYP
 
 
VAL
 
 
 
 
Weightbearing Forefoot Ab/Adduction
 
 
TYP
 
 
ABD
 
 
 
 
Weightbearing Foot Position
 
 
TYP
 
 
VAL
 
 
 
 
Weightbearing Midfoot Position
 
 
TYP
 
 
PLA
 
 
 
 
 
 
 
 
 
 
 
 
 
 
 
 
 
 
Measure
 
 
Left
 
 
Right
 
 
 
 
 
 
Non-Weightbearing Arch
 
 
TYP
 
 
TYP
 
 
 
 
Non-Weightbearing Forefoot Varus/Valgus
 
 
NEU
 
 
NEU
 
 
 
 
Non-Weightbearing Forefoot Ab/Adduction
 
 
NEU
 
 
NEU
 
 
 
 
Non-Weightbearing Hindfoot Eversion
 
 
TYP
 
 
TYP
 
 
 
 
Non-Weightbearing Hindfoot Inversion
 
 
TYP
 
 
TYP
 
 
 
 
Non-Weightbearing Hindfoot Varus/Valgus
 
 
VER
 
 
VER
 
 
 
 
Non-Weightbearing Midfoot Motion
 
 
HYP
 
 
HYP
 
 
 
 
First Ray Plantarflexion
 
 
TYP
 
 
TYP
 
 
 
 
Non-Weightbearing Subtalar Neutral
 
 
Y
 
 
Y
 
 
 
 
 
 
 
 
 
 
   
   Current    History Left    History Right   
 
 
     
 
 
     
 
 
     
 
 
 
 
 
 The plots show the development of various measurements with age. Measurement uncertainty, which may be substantial (e.g.,  \(\approx\pm 30^\circ\)  for Trochanteric Prominence Angle) is not shown. 
   
   Height & Weight    Femoral Anteversion & Tibial Torsion    Contracture    Motor Control, Mobility, and Gait    Crouch   
 
 
 
 
 
 
     
 
 
 
 
 
 
 
 
 
 
     
 
 
 
 
 
 
 
 
 
 
     
 
 
 
 
 
 
 
 
 
 
     
 
 
 
 
 
 
 
 
 
 
     
 
 
 
 
 
 
 
 
 
 
 
 
 Other Conditions 
 Typically barefoot vs. orthoses but may include other comparisons. 
   
  
 

 
 
 

 
 
 
   
 


  
